# GmCYP86A37 is a bifunctional cytochrome P450 essential for soybean root aliphatic suberin biosynthesis

**DOI:** 10.3389/fpls.2025.1744428

**Published:** 2026-01-28

**Authors:** Lorena S. Yeung, Andrea Ong, Sangeeta Dhaubhadel, Mark A. Bernards

**Affiliations:** 1 Department of Biology, Western University, London, ON, Canada; 2 London Research and Development Centre, Agriculture and Agri-Food Canada, London, ON, Canada

**Keywords:** aliphatic suberin, CYP86A, fatty acid ω-hydroxylase, fatty acid metabolism, soybean

## Abstract

18-Hydroxyoleic acid and its dioic acid derivative, oleic-1,18-dioic acid, are the two most prominent aliphatic monomers in soybean root suberin. While hydroxylated fatty acids are known to be formed by cytochrome P450 monooxygenases (P450), mainly from the CYP86A and CYP86B subfamilies, the biosynthetic origin of their corresponding dioic acids in soybean remains unclear. Two root-expressed soybean P450 genes, *GmCYP86A37* and *GmCYP86B9* were cloned and expressed as recombinant enzymes in yeast. A third root-expressed soybean P450 gene (*GmCYP86A38*) was also cloned, but no recombinant protein was produced. *In vitro* assays demonstrated that GmCYP86A37 and GmCYP86B9 exhibited preference for the ω-hydroxylation of oleic acid (C_18:1_) and lignoceric (C_24:0_) acids, respectively. Surprisingly, *in vitro* production of oleic-1,18-dioic acid was also detected when GmCYP86A37 was supplied with oleic acid substrate. Furthermore, CRISPR/Cas9-mediated double knockout of *Gmcyp86a37/38* resulted in substantial reduction of ω-hydroxylated fatty acids and dioic acids. These findings underscore the role of the CYP86A subfamily in soybean aliphatic suberin biosynthesis and provide direct evidence for GmCYP86A37 in the formation of oleic-1,18-dioic acid.

## Introduction

Suberin is a natural plant cell-wall biopolymer deposited in belowground tissues such as the root epidermis and endodermis. Structurally, suberin consists of both poly(phenolic) and poly(aliphatic) components, which together form a protective barrier against desiccation and pathogen invasion. In soybean (*Glycine max* [L.] Merr.), natural variation in root suberin levels has been associated with differential resistance to soil-borne pathogens (Thomas et al., 2007), with higher aliphatic suberin content conferring greater resistance. Accordingly, suberin is a promising target for crop improvement.

Omega (ω)-hydroxylation of fatty acids (FA) by cytochrome P450 (P450) enzymes is a major modification in suberin biosynthesis, with over half of aliphatic suberin monomers in plants being ω-hydroxylated FAs and/or α,ω-dicarboxylic acids (Holloway, 1983). Terminal carbon oxidation introduces additional functional groups (-OH and/or -COOH) enabling aliphatic suberin polymerization.

Several plant P450s of subfamily CYP86A function as FA ω-hydroxylases involved in suberin biosynthesis. In Arabidopsis, CYP86A1 catalyzes the ω-hydroxylation of a broad range of C_12–18_ saturated and unsaturated FAs, with a preference for palmitate (C_16:0_) to yield 16-hydroxypalmitic acid (Benveniste et al., 1998). Loss-of-function *Atcyp86a1* displayed marked reductions in C_16_-C_20_ ω-hydroxyacids and α,ω-dicarboxylic acids, along with ~60% lower total aliphatic suberin (Höfer et al., 2008). In potato, *CYP86A33* silencing by RNAi reduced C_18:1_ ω-hydroxyacids by ~70% and α,ω-dicarboxylic acids by up to 90% (Serra et al., 2009).

Arabidopsis CYP86B1 and CYP86B2 are root-expressed CYPs that share ~45% sequence identity with CYP86As. Although T-DNA mutants showed no obvious altered suberin phenotype, metabolic profiling of *Atcyp86b1* revealed sharp reductions in C_22_-C_24_ ω-hydroxyacids and α,ω-dicarboxylic acids, coupled with increases in their putative FA precursors (Compagnon et al., 2009). While no CYP86B enzyme has been biochemically characterized, the data support AtCYP86B1 as an ω-hydroxylase with very long chain fatty acid (VLCFA) specificity (Compagnon et al., 2009). A CYP86B homolog from rice has been shown to preferentially hydroxylate VLCFAs (Wassmann, 2014).

The biosynthesis of α,ω-dicarboxylic acids from ω-hydroxy FAs likely proceeds via sequential oxidation of the ω-carbon, first to an ω-oxo FA and then to an α,ω-dicarboxylic acid (see Woolfson et al., 2022 for a comprehensive overview of suberin monomer biosynthesis). Enzyme assays implicated putative NADP-dependent dehydrogenases in this process (Agrawal and Kolattukudy, 1978) in potato. In other species, single P450s have been reported to catalyze the three-step oxidation of the ω-carbon. *Vicia sativa* CYP94A1 has been shown to oxidize C_10–18_ FAs (saturated and unsaturated) and epoxy/mid-chain hydroxylated FAs to their α,ω-dicarboxylic acids counterparts *in vitro* (Tijet et al., 1998). *Nicotiana tabacum* CYP94A5 can ω-hydroxylate C_12–18_ FAs and showed activity toward 9,10-epoxystearic acid (Le Bouquin et al., 2001). Likewise, Arabidopsis CYP94C1 produced 12-hydroxylauric acid and α,ω-dodecadioic acid from lauric acid *in vitro* (Kandel et al., 2007). However, production of α,ω-dicarboxylic acids by members of other CYP subfamilies has not been reported.

Several soybean CYP86As have been partially characterized (Tully et al., 2020). Phylogenetic analysis of soybean FA ω-hydroxylases groups *Gm*CYP86A37 and *Gm*CYP86A38 with *At*CYP86A1 and *St*CYP86A33. Likewise, *Gm*CYP86B9 groups with known members of CYP86B (VLCFA ω-hydroxylase) from Arabidopsis and rice. Transcriptome data indicate highly specific expression of all three soybean P450s in roots and nodules. RNAi knockdown of *CYP86A37* and *CYP86A38* in soybean hairy roots led to a marked reduction in 18-hydroxyoleic acid without significant changes in total suberin (Tully et al., 2020), suggesting their roles in the terminal oxidation of oleic acid but also possible functional redundancy with other CYPs. While phylogenetic evidence points to CYP86B9 favoring VLCFA substrates, it may possess broader specificity that enables compensation under *CYP86A37/38*-RNAi knockdown. However, direct biochemical confirmation of substrate preferences for these soybean enzymes is still lacking. Soybean contains 16 CYP94A genes eight of which are expressed almost exclusively in roots with another two expressed in root tips and lateral roots (Khatri et al., 2022). No GmCYP94A genes have been functionally characterized.

In the present study, *in vitro* enzyme assays revealed *Gm*CYP86A37 and *Gm*CYP86B9 are functional FA ω-hydroxylases with broad substrate acceptance. GmCYP86A37 displayed the highest affinity for C_18:1_ and C_16:0_ FAs, whereas GmCYP86B9 preferentially hydroxylated C_24:0_ FA. Notably, we report the *in vitro* conversion of C_18:1_ oleic acid into both 18-hydroxyoleic acid and oleic-1,18-dioic acid by *Gm*CYP86A37. *In planta*, both 18-hydroxy oleic acid and oleic-1,18-dioc acid (Δ^9^-1,18-octadecenedioic acid) were reduced in *Gmcyp86a37*/*38* CRISPR soybean lines. Together, these results extend our functional understanding of CYP86 family enzymes and highlight their prospective potential in engineering crops with higher suberin content and improved resistance.

## Materials and methods

### Cloning & expression of soybean P450s

Soybean seeds (cv. Williams 82) were surface sterilized in 70% ethanol, rinsed five times with sterilized water and germinated in sterile vermiculite in 4L pots under controlled conditions (16 h-day/8 h-night; 26°C – 30°C). Roots of 14-day old soybeans were excised, flash frozen, and homogenized in liquid nitrogen. RNA was isolated from soybean roots using TRIzol™ Reagent (Invitrogen, USA), treated with DNase1 using TURBO DNA-*free*™ Kit (Thermo Fisher Scientific, USA) and reverse transcribed into cDNA using the Maxima First Strand cDNA Synthesis Kit (Thermo Fisher Scientific, USA), all according to manufacturer’s instruction.

Full-length *GmCYP86A37* (*Glyma.14G192500.1*)*, GmCYP86A38* (*Glyma.11G175900.1*) and *GmCYP86B9* (*Glyma.11G100100.1*) were amplified from soybean cDNA using gene-specific primers (**Supplementary Table 1**). Amplicons were cloned into pDONR/ZEO entry vector (Invitrogen, USA) using Gateway^®^ BP Clonase™ II Enzyme mix, transformed into *Escherichia coli* DH5α via CaCl_2_ heat-shock and grown on low-salt LB media supplemented with 50 μg/mL zeocin. Colony PCR was used to screen *E. coli* colonies, with successful transformants verified by Sanger Sequencing at the London Regional Genomics Center sequencing facility. Verified constructs were recombined into the destination vector pESC-Leu2d-LjCPR1-GW (Khatri et al., 2023), using LR Clonase™ II Enzyme mix (Invitrogen, USA). Recombinant plasmids were transferred into *E. coli* DH5α using CaCl_2_ heat-shock and screened by colony PCR.

Positive pESC-Leu2d-LjCPR1-GW-GmCYP86A/B plasmid DNA was transferred into *Saccharomyces cerevisiae* strain BY4742 by LiAc-heat shock transformation (Kawai et al., 2010). Colony PCR was performed on yeast colonies to confirm positive transformations.

### Heterologous protein expression

*S*. *cerevisiae* containing recombinant pESC-Leu2d-LjCPR1-GW-GmCYP86A/B was grown in 400 mL Leucine-Dropout_Glucose liquid media for 2 days at 30°C with agitation (200 rpm). Yeast cells were collected by centrifugation (6100 rcf) and washed twice with sterile water before resuspension in 1L Leucine-Dropout_Galactose (Leu-DO_Gal) liquid media containing 0.67% (w/v) yeast nitrogen base (Sigma-Aldrich, USA), 2% (w/v) D-Galactose (Sigma-Aldrich, USA), 1% (w/v) D-Raffinose (Sigma-Aldrich, USA), and 0.069% (w/v) Leucine-dropout supplement (Takara Bio Inc, USA). After 2 days, cells were collected as above and resuspended in 100 mL 100 mM potassium phosphate buffer (pH 7.6) containing 1% protease inhibitor.

Cells were lysed using a CF1 High-pressure homogenizer (Constant Systems Ltd, UK) set to 43 kpsi. Cell lysates were centrifuged at 10,000 rcf for 10 mins at 4°C and the microsome pellet collected by ultracentrifugation at 100,000 rcf for 70 mins at 4°C. Microsomes were resuspended in phosphate buffer (pH 7.6) and their protein concentration quantified using Bradford assay (Bio-Rad Laboratories, Inc, United States). Since the recombinant P450s harbor no epitope tags, their expression in yeast was verified by MS/MS–based sequencing at the BioCORE Protein Facility, Western University, Schulich School of Medicine and Dentistry, London, ON, Canada (**Supplemental Methods**).

### Fatty acid ω-hydroxylation enzyme assay

Fatty acid substrates C_16:0_, C_18:0_, C_18:1_, C_20:0_, C_22:0_, and C_24:0_ were chosen based on previous studies (Thomas et al., 2007; Tully et al., 2020) demonstrating soybean aliphatic suberin as predominantly composed of 16:0 and 18:1 ω-OH fatty acids and dioic acids, with minor contributions from 20:0, 22:0 and 24:0 ω-OH and dioic acids. Substrates dissolved in tetrahydrofuran were added to microsomal proteins (3 mg) in 50 mM phosphate buffer (pH 7.6) containing 1 mM NADPH and incubated for 30 min at 25°C. The reaction was quenched by addition of 10 μL 3 M HCl. Enzyme assay products were analyzed by GCMS after methylation and trimethylsilylation as described (Meyer et al., 2011). For enzyme kinetics, a range of substrate concentrations (5 μM, 10 μM, 20 μM, 50 μM, 100 μM, 200 μM for C_16:0_ and C_18:1_; 200 μM 400 μM, 600 μM, 800 μM, 1000 μM for C_18:0_, C_20:0_, C_22:0_, C_24:0_) was used. Product formation was confirmed by GCMS and comparison of mass spectra to the curated LipidWeb online resource (https://lipidmaps.org/resources/lipidweb/). Peak areas based on unique quant ions for each product were averaged (n = 5) and normalized to the triacontane internal standard. Lineweaver Burk plot x-intercepts were used to estimate *K_m_*.

### Chemotyping of cyp86a37/38 knockout lines for altered suberin

T1 seeds of CRISPR-Cas9 *Gmcyp86a37/38* knock out (KO) lines (cv. Williams 82) were obtained from the Wisconsin Crop Innovation Centre (University of Wisconsin Madison, Middleton, WI, USA), grown in soil under greenhouse conditions, and screened for altered root suberin phenotype. Briefly, several lateral roots were collected from 14-day old seedlings, flash frozen and homogenized using liquid nitrogen. Seedlings were re-planted and returned to the greenhouse. Approximately 20 mg of ground root tissue was Soxhlet-extracted, and the residue subject to aliphatic suberin analysis as described (Meyer et al., 2011). T1 plants showing altered suberin phenotype were grown to seed, and the T2 seed used for further analysis. Analysis of T2 seedlings was done as above, except that plants were initially grown in vermiculite and ground root tissue was extracted using MeOH-water-M*t*BE (Giavalisco et al., 2011). Target compound abundance was quantified based on specific quant ion peak area from the GC-MS, normalized to tissue weight and internal standard. The relative abundance of each target compound was compared to that in WT (n = 6). For each *Gmcyp86a37/38* KO line, 3 technical replicates were analyzed.

## Results

### Expression of GmCYP86A and GmCYP86B in yeast

All three soybean P450s, *GmCYP86A37*, *GmCYP86A38* and *GmCYP86B9* were successfully cloned and integrated into a yeast expression system (**Supplementary Figure 1**). However, only peptide sequences specific to GmCYP86A37 and GmCYP86B9 were identified in microsomes from yeast strains harboring the respective constructs following galactose induction, despite confirmed LjCPR1 expression in all yeast transformants (**Supplementary Figure 2**).

### GmCYP86A37 and GmCYP86B9 substrate specificity

**Figure 1:**
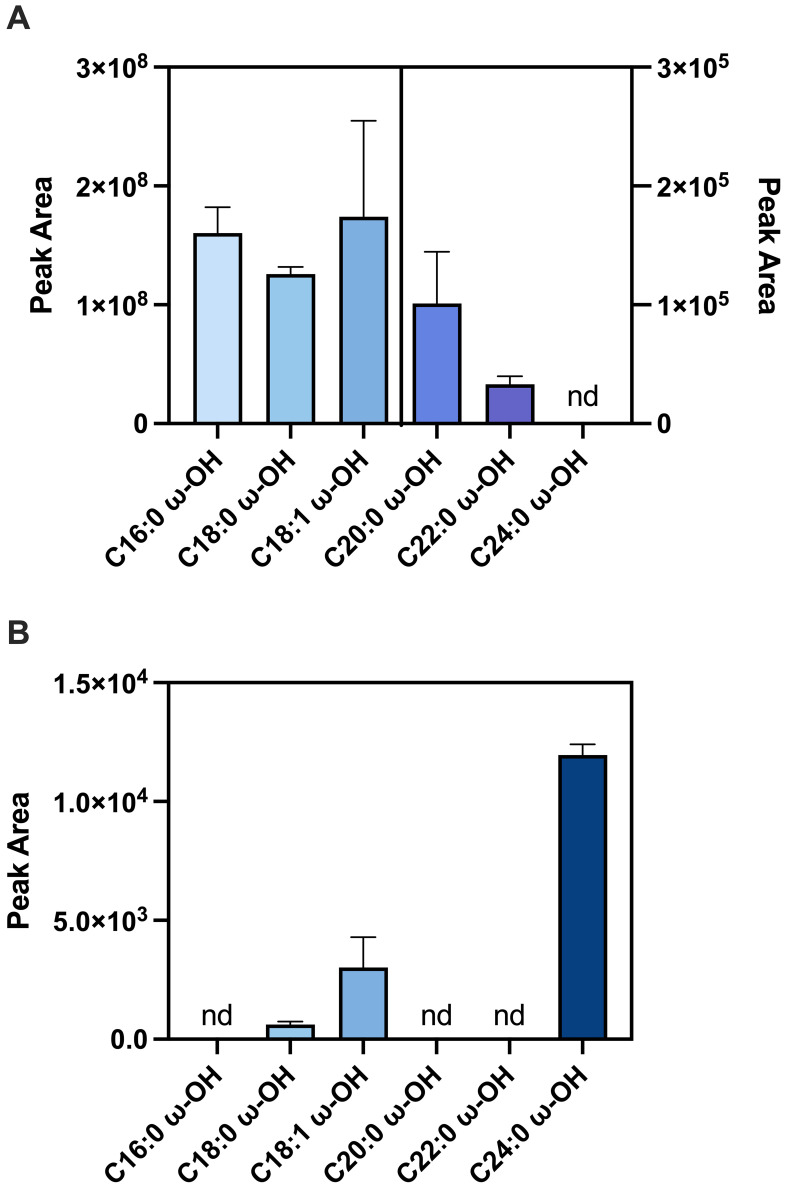
Substrate preference for recombinant GmCYP86A37 and GmCYP86B9. Recombinant GmCYP86A37 **(A)** and GmCYP86B9 **(B)** were incubated with FA substrates (200 mM) in the presence of NADPH and *L. japonicus* cytochrome P450 reductase, and the formation of ω-hydroxylated products assessed by GCMS. ω-Hydroxylated products were identified by comparing peak mass spectra to those at the curated LipidWeb database (https://lipidmaps.org/resources/lipidweb/). Peak area values are for product specific quantification ions (mean ± SEM) from triplicate (n = 3-4) assays. nd = not detected.

FA substrates ranging from C_16_-C_24_ were used with recombinant GmCYP86A37 and GmCYP86B9 *in vitro* at a final concentration of 200 mM. GmCYP86A37 showed preference for long chain fatty acids (C_16-18_), while its ability to hydroxylate VLCFA (C_20-24_) decreased with increasing hydrocarbon length (**Figure 1A**). No product formation was detected when C_24:0_ was used as substrate. By contrast, GmCYP86B9 preferentially hydroxylated C_24:0_ with very little or no activity toward the other substrates (**Figure 1B**).

### Enzyme Kinetics of GmCYP86A37 and GmCYP86B9

**Figure 2:**
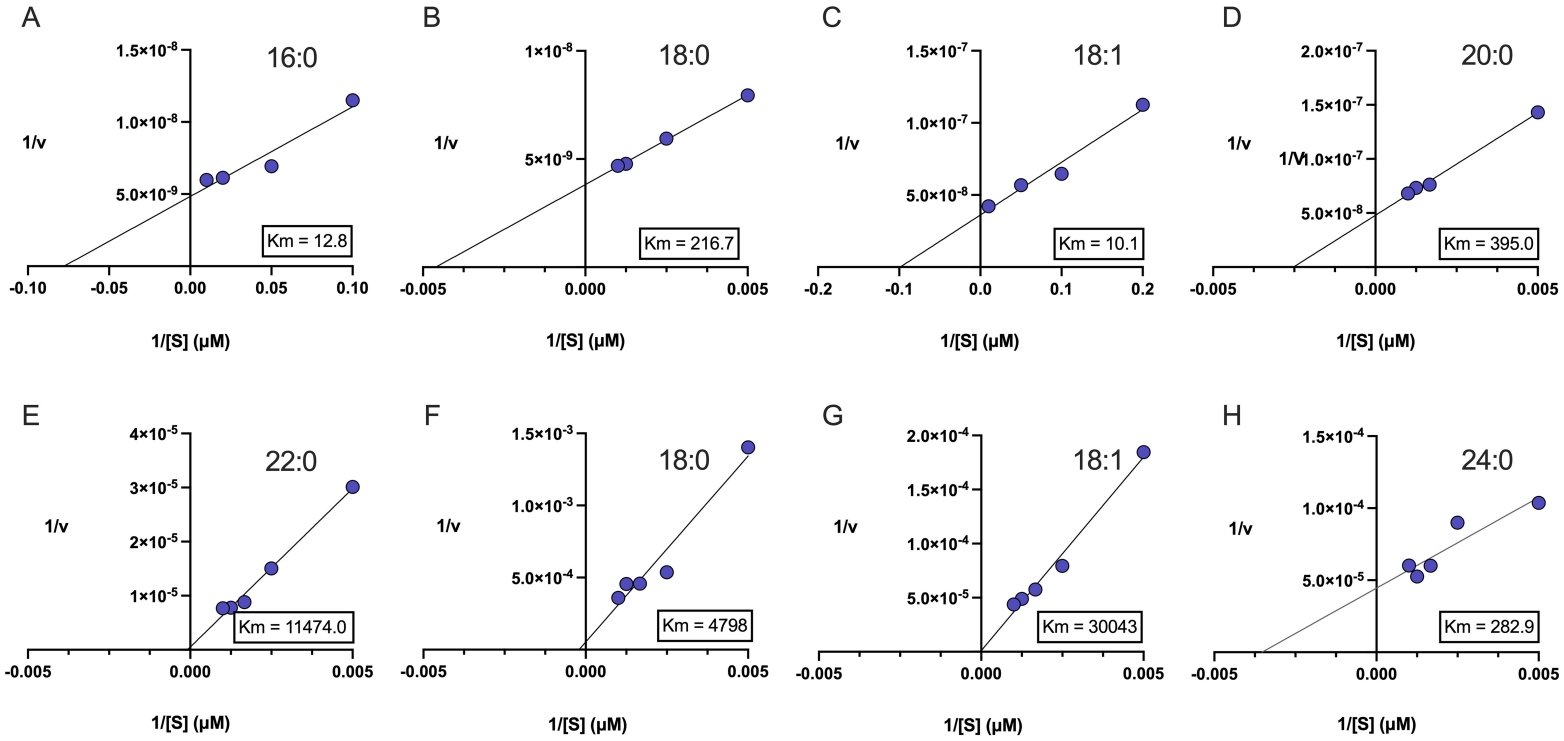
Kinetics of GmCYP86A37 and GmCYP86B9. Recombinant GmCYP86A37 and GmCYP86B9 were incubated with FA substrates in the presence of NADPH and *L. japonicus* cytochrome P450 reductase, and the formation of ω-hydroxylated products assessed by GCMS. ω-Hydroxylated products were identified by comparing peak mass spectra to those at the curated LipidWeb database (https://lipidmaps.org/resources/lipidweb/). Lineweaver-Burke plots were generated to estimate *K_M_* for each substrate. **(A-E)**, GmCYP86A37 incubated with **(A)** palmitic (C_16:0_), **(B)** stearic (C_18:0_), **(C)** oleic (C_18:1_), **(D)** arachidic (C_20:0_), and **(E)** behenic (C_22:0_) acids. **(F, G)**, GmCYP86B9 incubated with **(F)** stearic (C_18:0_), **(G)** oleic (C_18:1_), and **(H)** lignoceric (C_24:0_) acids. Five replicate assays were conducted (n = 5), with mean values plotted. All *K_M_* values are in mM.

For recombinant GmCYP86A37, *K_m_* estimates ranged from 10.1 mM for C_18:1_ FA to > 11 mM for C_22_ FA substrates (**Figures 2A–E**). By contrast, recombinant GmCYP86B9 displayed hydroxylation activity only toward C_18_, C_18:1_ and C_24_ FA substrates, with K_m_ estimates of 283 mM (C_24_), 4.8 mM (C_18_) and > 30 mM (C_18:1_), respectively (**Figures 2F–H**).

### GmCYP86A37 produces oleic-1,18-dioic acid *in vitro*

**Figure 3:**
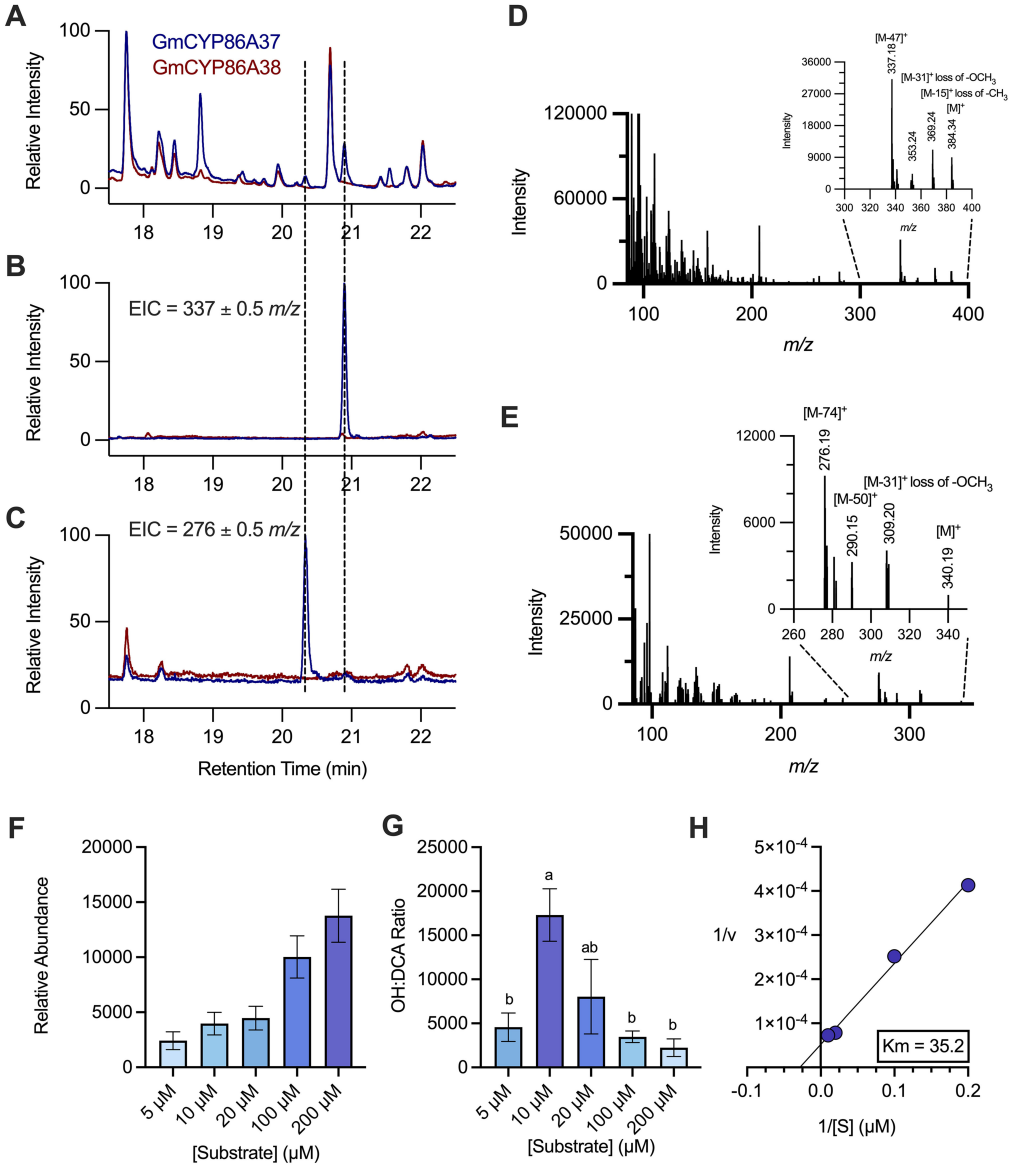
Formation of oleic-1,18-dioic acid by recombinant GmCYP86A37. Recombinant GmCYP86A37 was incubated with oleic acid (C18:1) in the presence of NADPH and *L. japonicus* cytochrome P450 reductase and the reaction products assayed by GCMS. **(A)** Total ion chromatogram (TIC) of reaction products from recombinant GmCYP86A37 and a negative control (recombinant GmCYP86A38). **(B, C)** Extracted ion chromatograms (EIC) of **(B)** m/z 337 ± 0.5 and **(C)** m/z 276 ± 0.5, representing 18-hydroxyoleic (as TMS-ether, methyl ester) and oleic-1,18-dioic (as dimethyl ester) acids, respectively. **(D, E)** Mass spectra obtained from recombinant GmCYP86A37 products at 20.9 **(D)** and 20.5 **(E)** minutes, matching 18-hydroxyoleic acid TMS-ether, methyl ester and oleic-1,18-dioic acid dimethyl ester, respectively. Inset spectra are enlargements of the diagnostic, high mass fragments of each compound. **(F)** Production of oleic-1,18-dioic acid over a range of oleic acid substrate concentrations. **(G)** Ratio of 18-hydroxyoleic and oleic-1,18-dioic acid peak areas over a range of oleic acid substrate concentrations. **(H)** Kinetics of oleic-1,18-dioic acid formation. The K_M_^app^ is in mM. For F and G, n = 3. Bars labelled with the same letter are not significantly different after analysis by one-way ANOVA followed by Tukey’s *post-hoc* test (P < 0.05).

GCMS chromatograms of products formed by recombinant GmCYP86A37 incubated with oleic acid (C_18:1_) revealed a distinct peak with mass a spectrum consistent with 18-hydroxyoleic acid (**Figures 3A, B, D**). Closer examination of the chromatograms identified an additional, unique peak at an earlier retention time (approximately 20.5 mins; **Figures 3A, C**), the mass spectrum for which corresponded to oleic-1,18-dioic acid-dimethyl ester (**Figure 3E**). Diagnostic ions included [M-74]^+^ (276 *m/z*) and [M-31]^+^ (309 *m/z*), and the parent ion [M]^+^ at 340 *m/z* (Bonaventure et al., 2004). No equivalent oleic-1,18-dioic acid peak was observed at 20.5 mins in the negative control (induced yeast harboring non-expressed GmCYP86A38; **Figures 3A, C**). Additionally, no dioic acid production was detected with other FA substrates, including 16-hydroxypalmitic acid. The relative abundance of oleic-1,18-dioic acid in GmCYP86A37 reaction products increased with increasing substrate concentration (**Figure 3F**) but was always several orders of magnitude lower than that of 18-hydroxyoleic acid (**Figure 3G**). The *K_m_^app^* for oleic acid oxidation to oleic-1,18-dioic acid was 35.2 mM (**Figure 3H**).

### cyp86a37/38 reduces accumulation of ω-hydroxy FA and α,ω-dicarboxylic acids in transgenic soybeans

**Figure 4:**
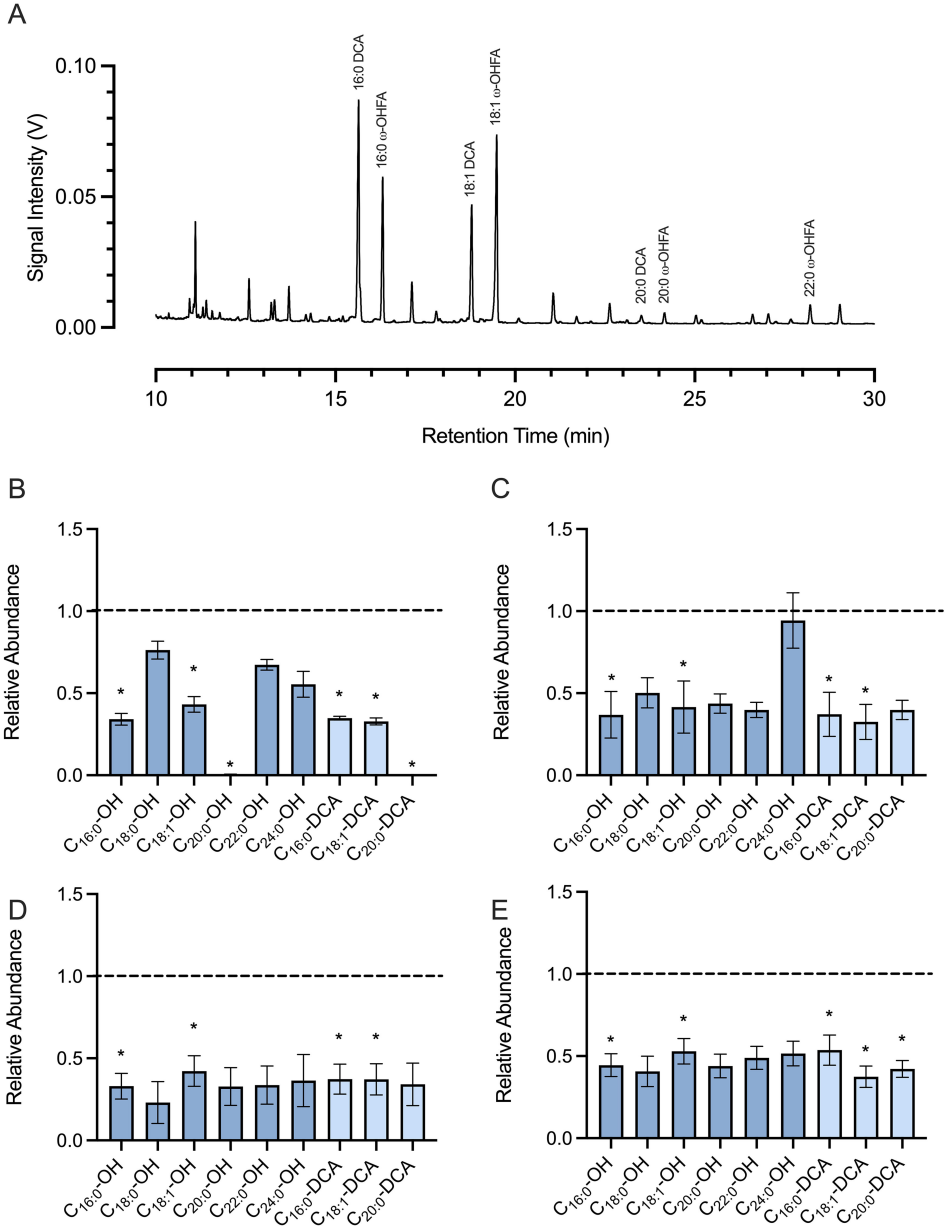
Oxidized aliphatic suberin monomers in select GmCYP86A37-KO/GmCYP86A38-KO lines. **(A)** Typical GCMS chromatogram of aliphatic suberin monomers isolated from soybean root suberin. Abundant oxidized (ω-OH, dioic) fatty acids are labelled. **(B–E)** Relative amounts of oxidized aliphatic suberin monomers in four independent *Gmcyp86a37/38* KO lines grown from T2 seed. Compounds were identified by comparing peak mass spectra to those at the curated LipidWeb database (https://lipidmaps.org/resources/lipidweb/). Peak areas for each aliphatic monomer were normalized to the amount of that monomer in wildtype samples (set to 1; dashed line). Bars labelled with an asterisk are significantly different from wildtype based on Student’s t-test (p < 0.05) comparing the mean peak area (n = 3) for each target compound.

While Gmcyp86a37/38 KO lines did not show any visible phenotype under greenhouse conditions, several, though not all T_2_ lines exhibited a marked reduction in aliphatic suberin monomer content (**Figure 4**). In these KO lines, the levels of long chain ω-hydroxy FA and α,ω-dicarboxylic acids (i.e., 16:0 and 18:1) were significantly decreased relative to wild type, whereas the levels of oxidized VLCFAs remained comparable to wild type levels.

## Discussion

*GmCYP86A37* and *GmCYP86B9* encode functional fatty acid ω-hydroxylases. GmCYP86A37 catalyzes formation of two predominant soybean aliphatic suberin monomers, 16-hydroxypalmitic acid and 18-hydroxyoleic acid, and additionally contributes to the accumulation of C_20_ and C_22_ ω -hydroxy FAs and oleic-1,18-dioic acid. In contrast, GmCYP86B9 primarily catalyzes ω-hydroxylation of VLCFAs, producing 24-hydroxylignoceric acid as a major product. The functional assignment of GmCYP86A37 as a fatty acid ω-hydroxylase was further supported by *in planta* analysis of *Gmcyp86a37/Gmcyp86a38* KO mutants. The substrate preference of GmCYP86A37 (18:1 > 16:0 > 18:0 > 20:0 > 22:0) aligns with prior characterization of its *Arabidopsis* homolog AtCYP86A1 (Benveniste et al., 1998), except GmCYP86A37 could also hydroxylate stearate (C_18:0_). Conversely, GmCYP86B9 showed strong preference for VLCFAs consistent with the reported substrate range of CYP86B homologs from rice (Wassmann, 2014) and Arabidopsis (Compagnon et al., 2009).

While the substrate preference of GmCYP86A37 to hydroxylate long chain FAs was expected, the formation of oleic-1,18-dioic acid from oleic acid (C_18:1_) was unanticipated. Indeed, this is the first demonstration of *in vitro* dioic acid formation by a CYP86A subfamily member as the formation of DCAs has been attributed to the CYP94 family, e.g., AtCYP94C1 (Kandel et al., 2007). With GmCYP86A37, dioic acid formation was specific to C_18:1_ with the three-step oxidation of oleic acid to oleic-1,18-dioic acid occurring within a single enzyme-substrate complex. Formation of 18-hydroxyoleic acid by GmCYP86A37 consistently exceeded that of oleic-1,18-dioic acid, indicating that 18-hydroxyoleic acid formation is preferred. The hydroxylated-to-dioic acid (OH:DCA) ratio was maximum (>15,000:1) with 10 μM oleic acid, corresponding to the substrate’s *K_m_* for the hydroxylation reaction. At higher substrate concentrations, the OH: DCA ratio decreased by several orders of magnitude, suggesting that dioic acid formation more readily occurs under conditions approaching substrate saturation (i.e., [S] > ten-fold higher than *K_m_*). GmCYP86A37 is therefore likely not the main biosynthetic enzyme for dioic acid formation in soybean, highlighting the need to characterize GmCYP94 family members.

We were unable to express recombinant GmCYP86A38 in yeast and could not assess its substrate preference or ability to form dioic acids. However, GmCYP86A37 and GmCYP86A38 share 79% identity at the amino acid level (Tully et al., 2020), and characterization of *Gmcyp86a37/38* RNAi knockdown lines only yielded a reduction in 18-hydroxyoleic acid in a hairy root system (Tully et al., 2020). It is unlikely that GmCYP86A38 contributes significantly to DCA production. Instead, the reduction in DCAs in *Gmcyp86a37/38* KO lines in the present study likely reflects a reduction in hydroxy fatty acids as substrates for GmCYP94A enzymes.

Our *in vitro* enzyme assays confirmed the molecular roles of GmCYP86A37 and GmCYP86B9 as functional fatty acid ω-hydroxylases and their ability to oxidize long-chain FAs and VLCFAs, respectively, *in vitro*. GmCYP86A37 was also uniquely capable of oxidizing oleic acid to oleic-1,18-dioic acid. Our data clarify the substrate specificities of suberin-associated CYPs and indicate that GmCYP86B9 exhibits overlapping substrate specificity with GmCYP86A37. This suggests a degree of functional redundancy in the suberin biosynthetic pathway and may explain why *Gmcyp86a37/38* knockdown (Tully et al., 2020) or knockout (herein) does not eliminate ω-hydroxy fatty acids from soybean root suberin.

## Data Availability

The original contributions presented in the study are included in the article/**Supplementary Material**. Further inquiries can be directed to the corresponding authors.
