# Supplementary material for: GmCYP86A37 is a bifunctional cytochrome P450 essential for soybean root aliphatic suberin biosynthesis
Source: Front Plant Sci. 2026 Jan 28;16:1744428. doi: 10.3389/fpls.2025.1744428 (PMC12891134; doi:10.3389/fpls.2025.1744428)
Supplement: Supplementary file 1 [file DataSheet1.pdf]

GmCYP86A37 is a bifunctional P450 essential for soybean root aliphatic suberin biosynthesis

Lorena S. Yeung[1,2], Andrea Ong[1], Sangeeta Dhaubhadel[1,3*] and Mark A. Bernards[1*]

Supplemental Data

Supplemental Methods: Induced Recombinant Protein Expression Verification by MS/MS Sequencing

CYP expression was verified by MS/MS based sequencing of expressed protein samples. Briefly, digestion buffer (1% SDC, 100 mM Tris-HCl, pH = 8.5) was added to 75 µg microsomal protein in microcentrifuge tubes, incubated in a thermomixer (800 rpm) at 60°C for 10 mins, followed by addition of DTT (100 mM) and further incubation in the thermomixer (800 rpm) at 60°C for 1 h. After cooling, iodoacetamide (IAA) was added, and the samples were incubated for a further 30 mins in darkness at room temperature. DTT was added to remove residual IAA. 100 ng/µL Trypsin was added and the samples were incubated overnight at 37°C. Peptides were recovered with addition of 10% TFA and ethyl acetate and centrifugation at 13,000 rcf for 3 mins at room temperature. The bottom aqueous layer was transferred to a fresh microcentrifuge tube and ultrapure water was added. Samples were prepared for MS/MS analysis using Oasis HLB 1cc 60 or 30 mg sorbent bed solid phase extraction (SPE) columns (Waters Corporation, USA). Columns were activated twice with methanol, followed by equilibration using 0.1% formic acid and loading of peptide samples. Columns were washed with 0.1% formic acid, air dried and peptides eluted with 70% acetonitrile. Peptide samples were dried under N_2 and submitted for MS/MS-peptide sequencing at the BioCORE Facility at Western University, Schulich School of Medicine and Dentistry London, ON, Canada.

Dried peptides were resuspended in 1% acetonitrile containing 0.1% formic acid (1% ACN, 0.1% FA) with shaking (1400 rpm) for 5 min, followed by sonication in an ultrasonic bath for 20 min. Reconstituted peptides were centrifuged at 21,000 rcf for 7 min at room temperature, and diluted 1:5 in 1% ACN, 0.1% FA in Waters Total Recovery® autosampler vials for injection. Peptide samples were injected onto a Waters Acquity UPLC M-Class system (Waters) coupled to a Q Exactive Plus hybrid quadrupole-Orbitrap mass spectrometer (ThermoFisher Scientific). Samples were trapped (5 µL min^{-1}, 5 min) on a Symmetry C18 Trap Column, 5 µm, 180 µm × 20 mm (Waters) using 99% Mobile Phase A (H_2O/0.1% FA), 1% Mobile Phase B (ACN/0.1% FA), then resolved on a Peptide BEH C18 Column, 130Å, 1.7 µm, 75 µm × 200 mm (Waters) at 35°C and a flow rate of 300 nL min^{-}

^{1} using a nonlinear gradient (1-7% B, 1 min; 7-23% B, 44 min; 23-35% B, 15 min; 35-98% B, 5 min). All liquid chromatography (LC) and mass spectrometry (MS) solvents used were of Optima LC/MS grade from Fisher Scientific.

The Q Exactive Plus mass spectrometer was controlled by Xcalibur software v4.0 (ThermoFisher Scientific). Data were acquired in data-dependent acquisition mode (DDA) using a FT/FT/HCD (Fourier Transform/higher-energy collision dissociation) Top 12 scheme. Survey scans (MS1) were acquired from *m/z* 375 to 1,500 at a resolution of 70,000 with AGC (automatic gain control) set to 3 e^{6} and a maximum injection time of 250 ms. Multiply charged peptide ions were isolated using a quadrupole isolation window of *m/z* 1.2 and were fragmented using HCD with a NCE (normalized collision energy) set to 25%. Fragment ion scans (MS2) were acquired at a resolution of 17,500 with AGC set to 2 e^{5} and a maximum injection time of 64 ms. Dynamic exclusion was set to 30 s, and lock mass ion was enabled at *m/z* 445.120025.

Raw data were processed using PEAKS 12 (Bioinformatics Solutions Inc.) and searched against a custom database generated from the yeast microsomal proteome (strain BY4742) that includes the protein sequence for LjCPR1, GmCYP86A37, GmCYP86A38, GmCYP86B9. Precursor and fragment ion mass tolerance was set to 10 ppm and 0.02 Da, respectively. Specific cleavage with trypsin was selected with a maximum of 2 missed cleavages, peptide length of 6-45 residues, fixed modification of carbamidomethylation (C), and variable modifications acetylation (N-term), deamidation (NQ), and oxidation (M), with a maximum of 2 variable modifications allowed per peptide. Peptide and protein false discovery rate (FDR) was set to 1% for database search.

Supplemental Data

Supplemental Table 1. Primer Sequences for Cloning of GmCYPs. Primers used for cloning of full-length coding region of GmCYPs in Gateway cloning. Forward and reverse primer containing attB1 and attB2 sites at the 5' end respectively for downstream recombinant BP reactions. Bolded nucleotide sequences represent start (ATG) and stop codons (TTA/ CTA/ TCA). Underlined sequences represent attB1 and attB2 sites.

Target Gene	Primer sequence (5' → 3')	T_m°C
CYP86A37<br>(Glyma.14G192500.1)	F:GGGGACAAGTTTGTACAAAAAAGCAGGCTTC**ATG**TTCCTCATTCTAACCAAGTTAATC	53.4
	R:GGGGACCACTTTGTACAAGAAAGCTGGGTC**TTA**AGGTGAGGTGGCAACC	55
CYP86A38<br>(Glyma.11G175900.1)	F:GGGGACAAGTTTGTACAAAAAAGCAGGCTTC**ATG**GTGCAAATGGATACACTCCA	56.7
	R: GGGGACCACTTTGTACAAGAAAGCTGGGTC**CTA**TGCAGACGTGGTGGCCTG	61.9
CYP86B9<br>(Glyma.11G100100.1)	F: GGGGACAAGTTTGTACAAAAAAGCAGGCTTC**ATG**ACCAACACCACCACC	55.2
	R: GGGGACCACTTTGTACAAGAAAGCTGGGTC**TCA**TTTTAATCCATCTTCCAAGTG	51.4

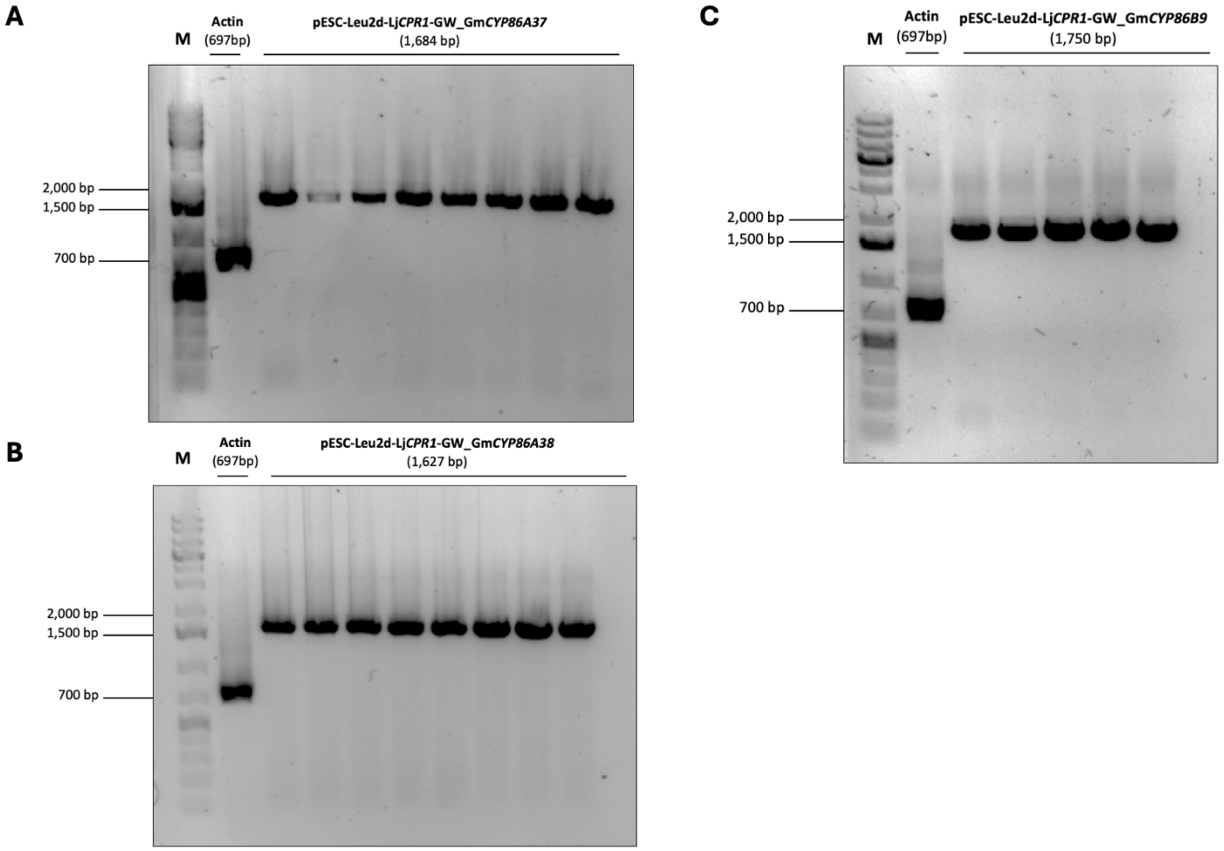

Supplemental Figure 1. Agarose gel analysis of pESC-Leu2d-LjCPR1-GW_GmCYP destination vector constructs. Colony PCR of yeast strain BY4742 transformed with destination vector pESC-Leu2d-LjCPR1-GW containing gene of interests: (A) GmCYP86A37 (2) GmCYP86A38 and (3) GmCYP86B9. PCR was run with gene-specific attB forward and reverse primers (see Supplemental Table 1). Actin was used as positive control. The expected band sizes for actin and the gene of interests are as listed. Each lane represents a different independently transformed yeast colony. M = molecular ladder with labelled reference bp sizes.

A >LjCPR1

1 MEESSSMKISPLDLMSAMIKGTLDPSNVSSTSGAGSVFLENREFVMVLTTSIAVLIGCVVVFIWRRSTGNKAKSIEPPKR

81 VVEKLSDEAEVDDGTRKVTIFFGTQTGTAEGFAKAIAEEAKVRYEKAKFKIVDMDDYAQDDDEYEEKLKKETLALFFLAT

161 YGDGEPTDNAARFYKWFLEGDEKEEGWLRNLEYAVFGLGNRQYEHFNKVAIEVDDKLADFGGKRLVKVGLGDDDQCIEDD

241 FTAWKEELWPALDELLRGDDDTTVSTPYTAAVLEYRVVIHDPLDASVDEKKWHNVNGHAIVDAQHPVRSNVAVRKELHTP

321 VSDRSCTHLEFDISGTGVAYETGDHVGVYCENLSETVEEAVRLLGLSPDTYFSVHTDDEDGKPLSGSSLPPTFPPCTLRT

401 AIARYADVLSSPKKSVLLALAAHASNPSEADRLRHLASPAGKDEYSEWVIASQRSLLEVMAEFPSAKPPIGVFFAAIAPR

481 LQPRFYSISSSPRMAPSRIHVTCALVNDKMPTGRIHRGVCSTWMKNSVPLEKSQDCSWAPIFVRQSNFKLPADNKVPIIM

561 IGPGTGLAPFRGFLQERLALKEDGAELGPSVLFFGCRNRQMDYIYEDELNHFVNSGALSELIVAFSREGPTKEYVQHKMM

641 EKASDIWNMISQGAYIYVCGDAKGMARDVHRTLHTILQEQGSLDSSKAEGMVKNLQLNGRYLRDVW

(continued)

B Glyma.14g192500.1. p (GmCYP86A37)

1 MFLILTKLIINVSYNTMMMQMETLPLLLTLVATLSAYFLWFHLLARTLTGPKPWPLVGSLPGLFRNRDRVHDWIADNLRG

81 RGGSATYQTCIIPFPFLARKKGFYTVTCHPKNLEHILKTRFDNYPKGPKWQTAFHDLLGQGIFNSDGETWLMQRKTAALE

161 FTTRTLKQAMSRWVNRSIKNRLWCILDKAAKERVSVDLQDLLLRLTFDNICGLTFGKDPETLSPELPENPFAVAFDTATE

241 ATMHRFLYPGLVWRFQKLLCIGSEKKLKESLKVVETYMNDAVADRTEAPSDDLLSRFMKKRDAAGSSFSAAVLQRIVLNF

321 VLAGRDTSSVALTWFFWLLTNHPDVEQKIVAEIATVLADTRGGDRRRWTEDPLDFGEADRLVYLKAALAETLRLYPSVPQ

401 DFKQAVADDVLPDGTEVPAGSTVTYSIYSAGRVETIWGKDCMEFKPERWLSVRGDRFEPPKDGFKFVAFNAGPRTCLGKD

481 LAYLQMKSVAAAVLLRYRLSLVPGHRVEQKMSLTLFMKNGLRVFLHPRKLESGPGVATSP

(continued)

C >Glyma.11g100100.1.p (GmCYP86B9)

1 MTNTTTSTILPFNQTNPMHSHIPFHSNTMTKPRNLNLTFLLQDIQILEIFLAVLVFIIIHSLRQKKHHGLAVWPVLGMVP

81 SLVTGLRTNLYEWITEVLKRQNGTFRFKGPWFSNLNCIVTSDPRNLEHLLKTKFPLYPKGGYFRNTVRELLGDGIFNADD

161 DTWQKQRKTASIEFHSTKFRQLTTESLFELVHYRLLPVLEASVKKSVAIDLQDILLRLTFDNVCMIAFGVDPGCLQLGLP

241 EIPFAKAFEDATEATVFRFVTPTCLWKAMKFLNLGMERKLNKSIKGVDEFAESVIRTRKKELSLQCEDSKQRLDLLTVFM

321 RLKDENGQAYSDKFLRDICVNFILAGRDTSSVALSWFFWLLEQNPQVEENILAEICKVVSQRKDIEREEFDNSLRFRPEE

401 IKKMDYLHAALSEALRLYPSVPVDHKEVVEDDTFPDGTVLKKGTKVIYAIYAMGRMEGIWGKDCKEFKPERWLRDGRFMS

481 ESAYKFTAFNGGPRLCLGKDFAYYQMKYAAASIVYRYHVKVVENHPVEPKLALTMYMKHGLKVNLYQRDAAQIQKHLEDG

561 LK

Supplemental Figure 2. Peptide alignment of trypsin-digested recombinant proteins. Total microsome was subjected to trypsin digestion. Sample clean-up was performed using SPE cartridges. Peptide sequencing was performed by BioCORE at Western University. Digested peptides were aligned to the yeast genome (strain: BY4742), *Lj*CPR1 sequence as well as *Gm*CYP sequences. Alignment was performed using sequences obtained from UniProt database. Underlines indicate coverage of peptides aligned to the target sequence, while grey highlights indicate the concensus sequences identified within the peptide mixtures. Coverage and sequence match for (A) a representative LjCPR1 sequence, (B) GmCYP86A37 and (C) GmCYP86B9 are shown. No peptides matching the GmCYP86A38 sequence were identified within the peptide mixture obtained from induced yeast harbouring the *pESC-Leu2d-LjCPR1-GW-GmCYP86A38* plasmid.
